# Targeting the HGF/c-MET pathway in advanced pancreatic cancer: a key element of treatment that limits primary tumour growth and eliminates metastasis

**DOI:** 10.1038/s41416-020-0782-1

**Published:** 2020-03-23

**Authors:** Zhihong Xu, Tony C. Y. Pang, Adele C. Liu, Srinivasa P. Pothula, Alpha Raj Mekapogu, Chamini J. Perera, Takashi Murakami, David Goldstein, Romano C. Pirola, Jeremy S. Wilson, Minoti V. Apte

**Affiliations:** 1 Pancreatic Research Group, South Western Sydney Clinical School, Faculty of Medicine, The University of New South Wales, Sydney, NSW Australia; 2 Ingham Institute for Applied Medical Research, Sydney, NSW Australia; 3 Faculty of Medicine, Saitama Medical University, Saitama, Japan

**Keywords:** Cancer microenvironment, Targeted therapies, Cancer models, Translational research

## Abstract

**Background:**

Stromal–tumour interactions facilitate pancreatic cancer (PC) progression. The hepatocyte growth factor (HGF)/c-MET pathway is upregulated in PC and mediates the interaction between cancer cells and stromal pancreatic stellate cells (PSCs). This study assessed the effect of HGF/c-MET inhibition plus gemcitabine (G) on the progression of advanced PC.

**Methods:**

Orthotopic PC was produced by implantation of luciferase-tagged human cancer cells + human PSCs into mouse pancreas. Tumours were allowed to develop without treatment for 4 weeks. Mice were then treated for 6 weeks with one of the following: IgG, G, HGF inhibitor (Hi), c-MET inhibitor (Ci), Hi + Ci, Hi + G, Ci + G, or Hi + Ci + G.

**Results:**

Bioluminescence imaging showed similar tumour sizes in all mice at the initiation of treatments. Triple therapy (Hi + Ci + G): (1) completely eliminated metastasis; (2) significantly reduced tumour size as assessed by bioluminescence and at necropsy; (3) significantly reduced proliferating cancer cell density and stem cell marker DCLK1 expression in tumours. In vitro 3D culture studies supported our in vivo findings.

**Conclusion:**

Even at an advanced disease stage, a two-pronged approach, targeting (a) HGF/c-MET with relevant inhibitors and (b) cancer cells with chemotherapy, completely eliminated metastasis and significantly decreased tumour growth, suggesting that this is a promising treatment approach for PC.

## Background

Pancreatic cancer is one of the few malignancies where the mortality is nearly equal to incidence, and is currently the fourth leading cause of cancer-related death in the United States.^1^ In the European Union, it was estimated to kill more patients than breast cancer and to be the third leading cause of cancer deaths in 2017.^2^ By 2030, pancreatic cancer is predicted to become the second leading cause of cancer-related death in United States.^3^ The lack of any significant improvements in the outcome of pancreatic cancer indicates that novel treatment strategies are urgently required.

Pancreatic cancer is characterised by its prominent desmoplastic/stromal reaction, which is composed of extracellular matrix proteins, such as collagen, fibronectin, proteoglycans, hyaluronan and stromal cells, including pancreatic stellate cells (PSCs), immune cells, endothelial cells and neuronal cells. This stroma plays an important role in cancer progression.^4^ PSCs are the principal source of stromal collagen and have been shown to promote primary tumour growth, induce resistance to chemo- and radiotherapy, and to facilitate metastasis.^5–7^ Cancer cells and PSCs interact with each other via a number of signalling molecules, such as vascular endothelial growth factor (VEGF), transforming growth factor-β (TGF-β), fibroblast growth factor-2 (FGF-2), platelet-derived growth factor (PDGF) and hepatocyte growth factor (HGF).^8–12^ PSCs also interact with endothelial cells via VEGF- and HGF-mediated signalling pathways.^7,13^ The expression of HGF and its receptor, c-MET, is upregulated in pancreatic cancer and associated with poor prognosis.^14,15^ Interestingly, PSCs produce and secrete HGF but do not express c-MET, while pancreatic cancer cells express c-MET but do not secrete HGF.^16^ The binding of HGF to its receptor c-MET is known to activate signalling pathways that modulate multiple cell functions, including proliferation, motility, migration and invasion. We have previously shown that mitogen-activated protein kinase (MAPK) and phosphatidylinositol 3-kinase (PI3K) are the major downstream signalling molecules that mediate HGF-induced proliferation and migration of cancer and endothelial cells.^11,13,16^

In our recently published pre-clinical study, we showed that treatment of very early orthotopic pancreatic cancer with HGF-c-MET inhibitors plus the chemotherapeutic agent gemcitabine (G) inhibited disease progression.^11^ However, the translational impact of these findings is limited, since only 15–20% of patients are diagnosed at an early stage of disease. Given that a majority of patients are diagnosed at an advanced stage, it would be essential to investigate the effect of HGF/c-MET inhibition in combination with gemcitabine in a model of advanced pancreatic cancer. This study employs just such a model involving implantation of human pancreatic cancer cell line AsPC-1 cells plus cancer-associated human PSCs into the mouse pancreas. Instead of initiating the treatment 1 week after implantation of cells [as was done by Pothula et al.,^11^] the tumours in this study were allowed to develop for 4 weeks (at which time tumours are well established and have metastasised) and the mice were then treated for 6 weeks with HGF/c-MET inhibitors and gemcitabine as single agent, or in dual or triple combinations. We found that the triple combination (HGF antibody + c-MET inhibitor + G) effectively reduced tumour volume and, more importantly, eliminated metastasis even at an advanced stage of the disease.

## Methods

### Orthotopic model of advanced pancreatic cancer (in vivo studies)

Luciferase-tagged human pancreatic cancer cell line AsPC-1 was provided by Dr. Murakami. Human PSCs were isolated using outgrowth method as described previously.^7,17^ The orthotopic model was established as described previously.^7,11,16^ Briefly, athymic mice (BALBc nu/nu) were anaesthetised and the pancreas was exteriorised following a left flank incision. A mixture of human pancreatic cancer cells (Luc-AsPC-1) 1 × 10^6^ + human PSCs 1 × 10^6^ in 50 μL phosphate-buffered saline (PBS) was injected into the pancreas. Organs were then replaced, and the abdomen was closed.

Luciferin was administered by intraperitoneal (IP) injection to monitor tumour burden in mice using IVIS Lumina II. Four weeks after implantation of Luc-AsPC-1 + human PSCs, mice were randomised to receive treatment. [Note: Four weeks was selected as the optimal time point representing advanced disease because our previous experience with this model has demonstrated that at 4 weeks, primary tumours are well established, and the cancer has metastasised. Furthermore, initiation of treatment at a later time would mean that the untreated (control) mice would have too big a tumour burden to survive the duration of the experiment (a situation unacceptable to our Animal Ethics Committee).^11^] Details of the treatment groups are as follows (*n* = 6 mice per group):

*IgG*: Isotype IgG IP twice weekly and soybean oil (vehicle control for c-MET inhibitor) daily oral gavage.

*G*: Gemcitabine 75 mg/kg IP twice weekly.

*Hi*: HGF inhibitor (Rilotumumab/AMG102 is a human HGF-neutralising antibody from Amgen Inc., Thousand Oaks, CA, USA) 300 μg in 200 μl PBS IP twice weekly; dose published before.^11,16^

*Ci*: c-MET inhibitor (compound A is a small-molecule inhibitor that blocks the ATP-binding pocket on c-MET, thus preventing activation, manufactured by Amgen Inc.) 60 mg/kg dissolved in soybean oil administered as daily oral gavage; dose published before.^11^

*Hi* *+* *Ci*: HGF antibody + c-MET inhibitor.

*Hi* *+* *G*: HGF antibody + G.

*Ci* *+* *G*: c-MET inhibitor + G.

*Hi* *+* *Ci* *+* *G or triple therapy*: HGF antibody + c-MET inhibitor + G.

Tumour growth was monitored by palpation and bioluminescent imaging. Mice were euthanised after 6 weeks of treatment (see Supplementary Methods, p 4, para 3 for details of euthanasia). Tumours were resected and measured using a digital Vernier calliper. Tumour volume was calculated according to an established formula [1/2 (length × breadth × width)].^6,7^ The abdominal cavity, mesentery, spleen, liver and lungs were assessed for metastases and verified by haematoxylin and eosin staining. To quantify metastasis metastatic score was calculated as previously published by us^11^: number of visible nodules per site (liver, mesentery, diaphragm, retroperitoneum and lung) × number of mice and expressed as % of control score in IgG-treated mice. Tumours and metastases were further analysed as described below.

### Tumour fibrosis and expression of α-SMA cytokeratin, PCNA, DCLK1, TUNEL, E-cadherin and vimentin

Fibrosis/collagen deposition was assessed by Sirius Red staining as described previously.^6,7^ Paraffin sections of tumours were incubated with respective primary antibodies against human α-SMA (α-smooth muscle actin) (1:800), cytokeratin (1:75), PCNA (proliferating cell nuclear antigen 1:200), Ki-67 (1:333), DCLK1 (1:500), E-cadherin (1:200) and vimentin (1:3000), followed by incubation with relevant secondary antibodies and DAB visualisation. TUNEL (terminal deoxynucleotidyl transferase dUTP nick-end labelling) staining was performed using In Situ Cell Death Detection Kit.

Morphometric analyses were performed on ten random fields (by observers blinded to experiments) of each section and positively stained (brown) cells were counted as described previously.^11^

### 3D organotypic culture (in vitro studies)

An in vitro three-dimensional (3D) organotypic culture was employed to further investigate the mechanisms of treatments observed on cancer progression. This consisted of a collagen matrix containing PSCs with cancer cells seeded on the top of the matrix. Collagen for the matrix was extracted from the tendons of rat tails, solubilised in 0.5 M acetic acid and precipitated by sodium chloride. The precipitated pellet was dissolved in 0.25 M acetic acid and dialysed in 17.4 mM acetic acid. The resulting solution was then centrifuged and the supernatant containing collagen was collected and diluted to 2 mg/mL with 17.4 mM acetic acid. Purity of collagen I was assessed by comparison with commercial products (BD Biosciences) using electrophoresis and staining.

Human PSCs were harvested, counted and resuspended in foetal bovine serum, and then mixed with 10× minimum essential medium, 0.22 M NaOH and collagen. The mixture was dispensed into 6-well plate and incubated at 37 °C and 5% CO_2_ allowing the collagen to polymerise. The density of human PSCs was adjusted to 500,000 cells per matrix. The matrix was submerged in the culture medium to contract over 7 days and transferred into 24-well plates when its size matched the well size.

AsPC-1 cells (300,000) were seeded on top of each matrix in co-culture medium (SFM4MAb®). After 7 days, the matrices were moved onto an elevated metal mesh. PSC culture medium was fed from the bottom of matrices and changed twice per week. IgG, HGF-neutralising antibody at 60 µg/mL and/or c-MET inhibitor (PHA-665752, from Tocris Bioscience) at 100 nM were added to the PSC medium. At the end of the incubation, matrices were fixed and paraffin embedded for subsequent immunochemistry.

Sections were incubated with primary antibodies (PCNA 1:400, caspase-3 1:200, caspase-9 1:250, ALDH-1 1:250, E-cadherin 1:100, vimentin 1:800 and cytokeratin 1:60) and relevant secondary antibodies. Antigen–antibody binding was visualised using DAB.

### Statistical analysis

Data are expressed as mean ± SEM. One-way analysis of variance with Tukey’s post hoc test was applied. Analyses were performed using GraphPad Prism 8 for Mac OS X (GraphPad Software, San Diego, CA, USA).

## Results

### Orthotopic model of advanced pancreatic cancer (in vivo studies)

#### Bioluminescence imaging

**Fig. 1:**
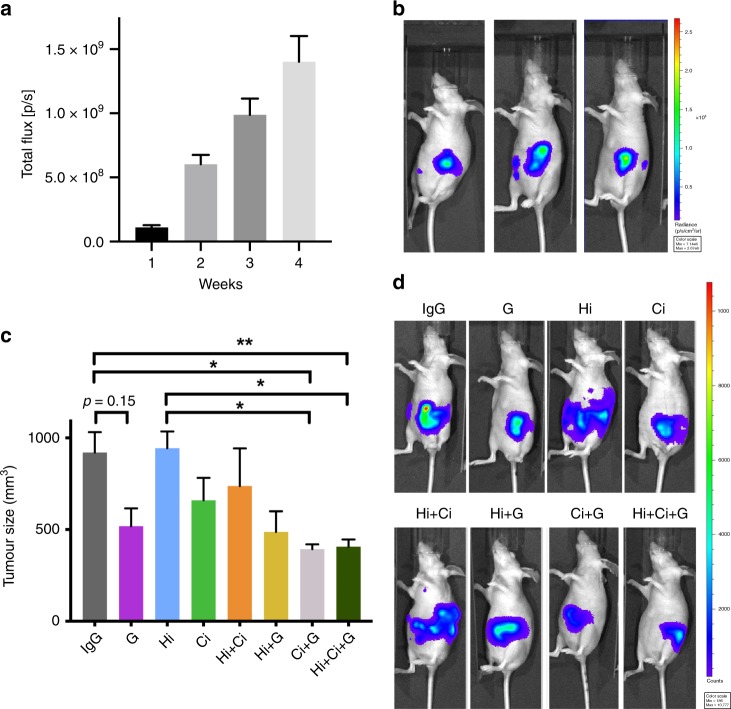
Primary tumour volume. **a** Bioluminescence imaging of mice over the first 4 weeks after cell implantation demonstrated that tumours grew at similar rates over that period, and importantly, that the tumour sizes were similar in all mice at the initiation of treatment. **b** Representative photographs of bioluminescent imaging at 4 weeks, showing that cancer cells have metastasised (luminescent signal separate from primary tumour). **c** Effects of HGF/c-MET inhibition ± gemcitabine on tumour volume. The combination of c-Met inhibitor + gemcitabine (Ci + G) and HGF-neutralising antibody + c-MET inhibitor + gemcitabine (triple therapy, Hi + Ci + G) significantly reduced tumour size. **p < 0.01 Hi + Ci + G vs. IgG; *p < 0.05 Hi + Ci + G vs. Hi, or Ci + G vs. IgG, or Ci + G vs. Hi; *n* = 6 mice per group. **d** Representative photographs of bioluminescent imaging at the end of the 6-week treatment period, showing that triple therapy (Hi + Ci + G) and Ci + G significantly inhibited tumour growth, as determined by both signal intensity and area. Spectrum ranges from weak (blue) to strong (red).

After implantation of cells into the pancreas, bioluminescence imaging showed linear growth before treatment, with little variation between mice at each time point (Fig. 1a). Thus, at the initiation of treatments, tumour size was similar in all mice. At the fourth week, bioluminescent images revealed signals distinct from primary tumour, indicating that cancer cells had metastasised (Fig. 1b), a scenario that closely resembled the clinical setting for most patients at diagnosis.

#### Effect of HGF/c-MET pathway inhibition ± G on tumour volume

Triple therapy (Hi + Ci + G) and dual treatment with Ci + G significantly decreased tumour size (Fig. 1c). Although tumours in mice treated with gemcitabine or Ci alone, or with dual combinations Hi + G or Hi + Ci were smaller than those in IgG-treated controls, the changes were not statistically significant. Notably, Hi alone had no effect on tumour growth in this advanced pancreatic cancer model. Representative bioluminescent images at the end of the treatment period are depicted in Fig. 1d.

#### Effect of HGF/c-MET pathway inhibition ± G on metastasis

**Fig. 2:**
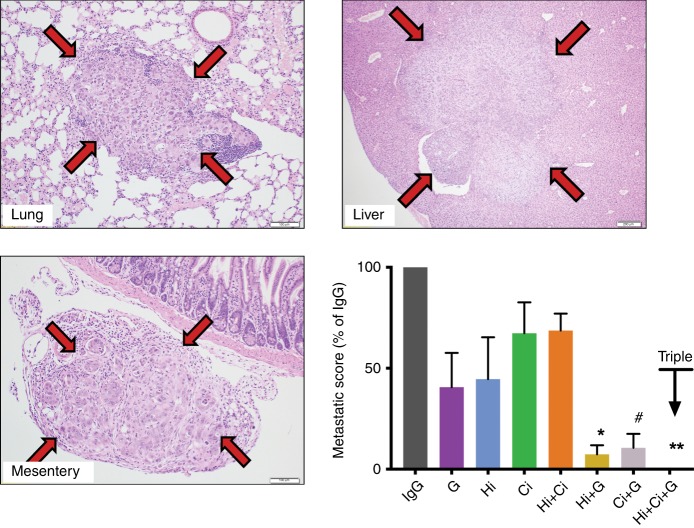
Effects of HGF/c-MET inhibition ± gemcitabine on metastasis. Representative photomicrographs of metastatic nodules in lung, liver and mesentery, as demarcated by the arrows. The bar graph shows the analysis results that the combination of HGF-neutralising antibody + gemcitabine (Hi + G) and c-MET inhibitor + gemcitabine (Ci + G) significantly reduced the incidence of metastasis compared to IgG controls. Notably, no metastases were detected in mice treated with HGF-neutralising antibody + c-MET inhibitor + gemcitabine (triple therapy, Hi + Ci + G). No significant effect was overserved with other treatment groups. **p < 0.01, Hi + Ci + G vs. IgG or Hi + Ci; *p < 0.05, Hi + G vs. IgG or Hi + Ci; ^#^*p* < 0.05 Ci + G vs. IgG; *n* = 6 mice per group.

G, Hi or Ci administered as single agents and the combination of Hi + Ci failed to reduce metastasis. However, treatment with Hi + G, Ci + G or triple therapy (Hi + Ci + G) significantly inhibited cancer metastasis compared to IgG. Particularly intriguing was the finding that mice treated with triple therapy had a complete absence of metastases as assessed by gross morphology as well as histological examination of extrapancreatic organs (Fig. 2 and Table 1).

**Table 1:** Number of mice per group with detectable metastases in different sites (*n* = 6 mice per group).

	IgG	G	Hi	Ci	Hi + Ci	Hi + G	Ci + G	Hi + Ci + G
Liver	6	2	5	3	4	1	0	0
Mesentery	5	1	2	1	3	1	1	0
Diaphragm	2	1	2	2	1	0	0	0
Retroperitoneum	1	1	0	1	1	0	0	0
Lung	3	0	0	2	2	0	1	0
Total	17	5	9	9	11	2	2	0

### Histological and immunohistochemical characterisation of primary tumours

#### PSC activation and collagen content

PSC activation was not influenced by the treatments (Supplementary data). Sirius Red staining showed that collagen deposition in tumours treated with gemcitabine alone was greater than any other group, although this increase did not reach statistical significance. Interestingly, tumours in mice treated with Hi + Ci exhibited a modest but significant reduction in collagen deposition compared to gemcitabine (Supplementary Fig. 1A, B).

#### Cancer cell numbers

Immunostaining for the cancer cell marker cytokeratin revealed that all treatments containing gemcitabine (G, Hi + G, Ci + G and Hi + Ci + G) significantly decreased cancer cell density in the tumours compared with other treatments (IgG, Hi, Ci and Hi + Ci) (Supplementary Fig. 2A, B).

#### Cancer cell proliferation

**Fig. 3:**
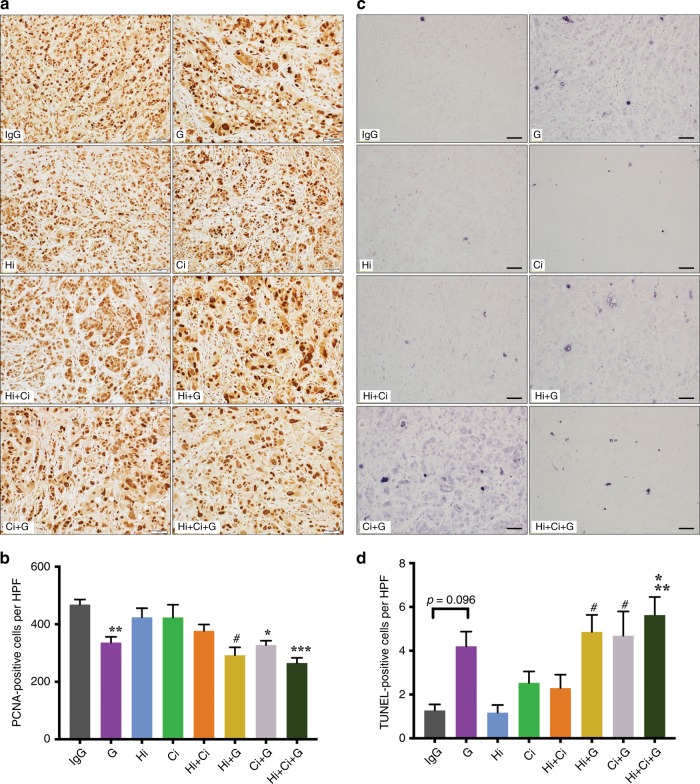
Effects of HGF/c-MET inhibition ± gemcitabine on cancer cell proliferation and apoptosis. **a** Representative photomicrographs of immunostaining for PCNA (brown nuclear staining). Scale bars = 50 µm. The negative control for PCNA staining (sections stained with equivalent concentration of isotype IgG) is shown in Supplementary Fig. 8a. **b** Effects of HGF/c-MET inhibition ± gemcitabine on cancer cell proliferation. Treatment with gemcitabine (G), HGF-neutralising antibody + gemcitabine (Hi + G), c-MET inhibitor + gemcitabine (Ci + G) or triple therapy (Hi + Ci + G) significantly reduced PCNA-positive cells (proliferating cells) in the tumours. ***p < 0.001, Hi + Ci + G vs. IgG, Hi or Ci; **p < 0.01, G vs. IgG; *p < 0.05 Ci + G vs. IgG; ^#^*p* < 0.05, Hi + G vs. IgG or Hi; *n* = 6 mice per group. **c** Representative photomicrographs of TUNEL staining (blue nuclear staining). Scale bars = 50 µm. The negative control and positive control for TUNEL staining are shown in Supplementary Fig. 8b, c, respectively. **d** Effects of HGF/c-MET inhibition ± gemcitabine on cancer cell apoptosis. The apoptotic cancer cell density in gemcitabine (G)-treated tumours showed a trend towards an increase compared to those treated with IgG (*p* = 0.096). Triple therapy (Hi + Ci + G) significantly induced cancer cell apoptosis in tumours compared to tumours in the IgG, HGF-neutralising antibody (Hi) and HGF-neutralising antibody + c-MET inhibitor (Hi + Ci) groups. HGF-neutralising antibody + gemcitabine (Hi + G) and c-MET inhibitor + gemcitabine (Ci + G) also significantly increased cancer cell apoptosis compared to IgG and Hi groups. **p < 0.01, Hi + Ci + G vs. IgG or Hi; *p < 0.05, Hi + Ci + G vs. Hi + Ci; ^#^*p* < 0.05, Hi + G or Ci + G vs. IgG or Hi; *n* = 6 mice per group.

Proliferating cancer cells were identified by PCNA. HGF/c-MET inhibition (Hi, Ci or Hi + Ci) did not alter cancer cell proliferation. However, significantly reduced cell proliferation was observed in all treatment groups involving gemcitabine compared to IgG (Fig. 3a, b). Similar trends were observed with another cell proliferation marker, Ki-67 (Supplementary Fig. 3A, B).

#### Cancer cell apoptosis

Apoptosis of cancer cells was assessed by TUNEL staining. Although treatment with gemcitabine alone showed a trend towards increased cancer cell apoptosis, this effect was not statistically significant. In contrast, Hi + Ci + G, Ci + G and Hi + G significantly increased cancer cell apoptosis compared to IgG- and Hi-treated mice. The number of apoptotic cancer cells in the Hi + Ci + G group was significantly higher than that in Hi + Ci mice (Fig. 3c, d).

#### EMT of cancer cells

**Fig. 4:**
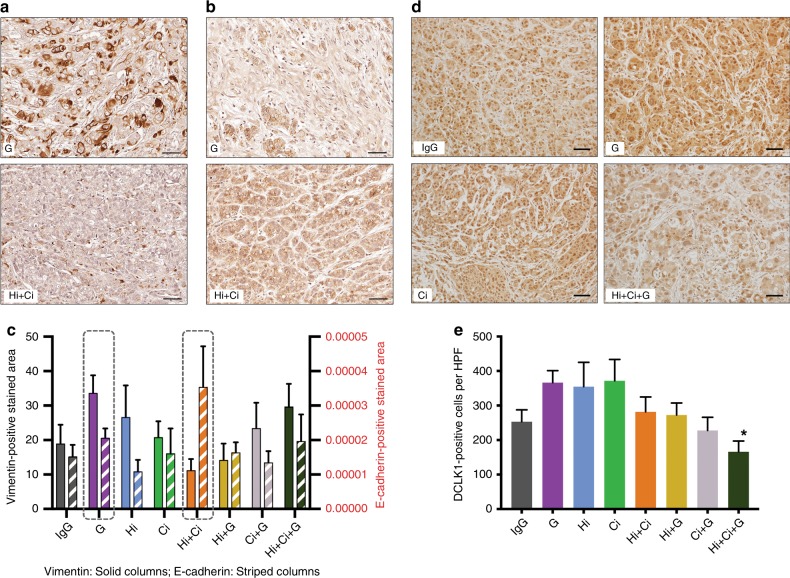
Effects of HGF/c-MET inhibition ± gemcitabine on cancer cell EMT and cancer stemness. **a** Representative photomicrographs of immunostaining for vimentin (dark brown) of G (gemcitabine) and Hi + Ci (inhibition of HGF and c-MET) groups. The full panel of representative images is allocated in Supplementary Fig. 4A. Scale bars = 50 µm. The negative control for vimentin staining is shown in Supplementary Fig. 8d. **b** Representative photomicrographs of immunostaining for E-cadherin (brown cell membrane staining) of G (gemcitabine) and Hi + Ci (inhibition of HGF and c-MET) groups. The full panel of representative images is allocated in Supplementary Fig. 4B. Scale bars = 50 µm. The negative control for E-cadherin staining is shown in Supplementary Fig. 8e. **c** Effects of HGF/c-MET inhibition ± gemcitabine on cancer cell EMT. The general pattern of vimentin and E-cadherin expression was that vimentin was higher than E-cadherin, except for the Hi + Ci Group (HGF-neutralising antibody + c-MET inhibitor). Comparing the expression for these two markers in the gemcitabine (G) and the HGF-neutralising antibody + c-MET inhibitor (Hi + Ci)-treated groups (highlighted by the dashed rectangles), it was observed that the expression of vimentin was high in G, but low in Hi + Ci. In contrast, E-cadherin expression was low in G, but high in Hi + Ci group. These data suggest that gemcitabine alone induces EMT, while inhibition of both HGF and c-MET protects from EMT. *n* = 6 mice per group. **d** Representative photomicrographs of immunostaining for DCLK1 (stem cell marker) of IgG, G (gemcitabine), Ci (c-MET inhibitor) and Hi + Ci (Inhibition of HGF and c-MET) groups. The full panel of representative images is allocated in Supplementary Fig. 5. Scale bars = 50 µm. The negative control for DCLK1 staining is shown in Supplementary Fig. 8f. **e** Effects of HGF/c-MET inhibition ± gemcitabine on cancer stemness. Triple therapy (Hi + Ci + G) significantly reduced DCLK1 expression in tumours compared to that in the gemcitabine (G) and c-MET inhibitor (Ci) treated groups. *p < 0.05, *n* = 6 mice per group.

A decrease in the epithelial marker E-cadherin and a concomitant increase in the mesenchymal marker vimentin is usually interpreted as indicative of the process of epithelial–mesenchymal transition (EMT). As assessed by immunohistochemistry, vimentin expression (representative images in Fig. 4a, full panel in Supplementary Fig. 4A) was decreased and E-cadherin expression (representative images in Fig. 4b, full panel in Supplementary Fig. 4B) was elevated in the Hi + Ci group compared to the other groups. Tumours of mice treated with gemcitabine alone showed the highest vimentin expression of all groups, although the increase did not achieve statistical significance (representative images in Fig. 4c, full panel in Supplementary Fig. 5). These data suggest gemcitabine alone induced EMT, while inhibition of both HGF and c-MET (Hi + Ci) protected from EMT.

#### Cancer stemness

Immunostaining for the stem cell marker DCLK1 demonstrated that G, Hi and Ci elevated DCLK1 expression compared to IgG, although this change did not achieve significance. Triple therapy resulted in a significant decrease in DCLK1 expression compared to the mice treated with gemcitabine alone (Fig. 4d, e), suggesting that cancer stemness may be inhibited by the combination treatment.

### 3D organotypic culture (in vitro studies)

3D organotypic cultures were performed to investigate the effects and mechanisms mediating the response of cancer cells to the above treatments.

Our previous studies^11,16^ have shown that cancer cell proliferation induced by human PSC secretions could be prevented by adding an HGF-neutralising antibody to the conditioned medium. These studies were carried out using a traditional 2D culture set-up. In the present study, we have applied an improved method that takes into account the possible influence of the extracellular matrix on cancer cell behaviour.

#### Cancer cell proliferation in vitro

This was assessed by staining matrices containing cancer cells and PSCs for PCNA. Gemcitabine alone or in dual or triple combinations with HGF/c-MET inhibition significantly reduced proliferating cancer cells compared with the rest of treatments (Supplementary Fig. 6A, B).

#### Cancer cell apoptosis in vitro

TUNEL staining showed that exposure of matrices to Hi + Ci, Hi + G, Ci + G or Hi + Ci + G significantly increased cancer cell apoptosis (Supplementary Fig. 7). The increased apoptosis with triple therapy (Hi + Ci + G) revealed by TUNEL staining was supported by findings of increased caspase-3 and -9 expression in this group. Caspase-3 was significantly increased by the treatment of G, HGF-neutralising antibody (Hi) alone, HGF-neutralising antibody + G (Hi + G) and triple therapy (G* 87.13 + 2.79%, Hi* 86.39 + 2.51%, Hi + G** 89.52 + 2.87%, Hi + Ci + G** 89.5 + 2.26% vs. IgG 75.13 + 2.85% *n* = 5, *p < 0.05, **p < 0.01). Caspase-9 expression was also statistically significantly induced in the Hi + Ci + G group (89.8 + 2.47% vs. IgG 80.29 + 3.64%, *n* = 5, *p* < 0.05 by analysis of variance Dunnett’s test).

#### Cancer cell migration, stemness and EMT

Cancer cell functions such as migration, stemness and EMT were assessed in the collagen matrices upon exposure to HGF-neutralising antibody (Hi), or c-MET inhibitor (Ci) alone or in combination for longer periods of 4 weeks. This extended incubation was assessed because studies by our groups and others had demonstrated that changes in these particular cell functions are not detectable after short incubations. Notably, gemcitabine was not used in this setting, because our preliminary studies had demonstrated that extended exposure to gemcitabine in the 3D setting resulted in a complete loss of cancer cells on the top of the matrices.

**Fig. 5:**
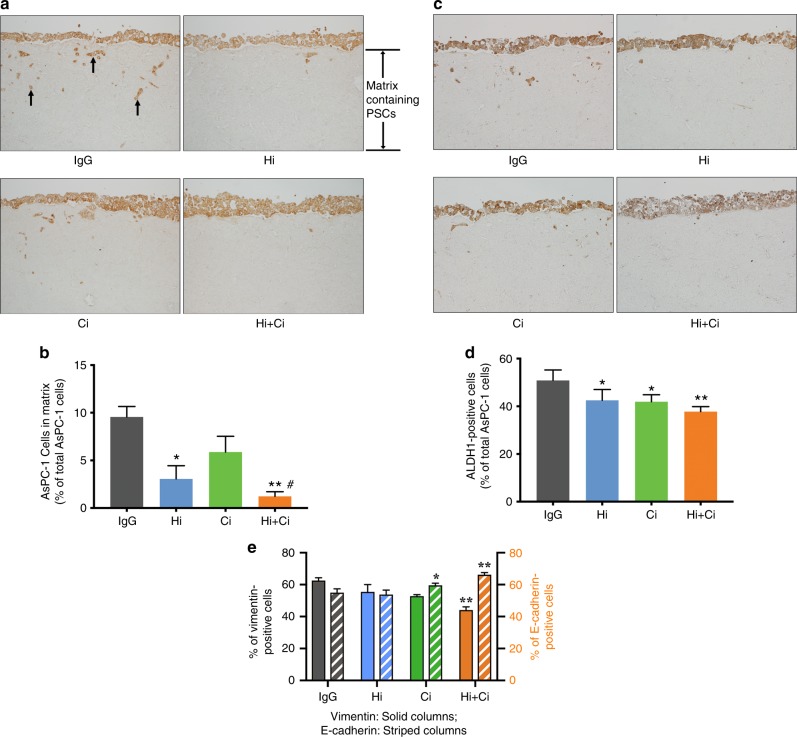
Effects of HGF/c-MET inhibition on cancer cell migration, stemness and EMT in vitro. **a** Representative cross sections of matrices were stained for cancer cell marker cytokeratin (brown). Cancer cells are on the top of the matrices and invade into the collagen matrix. Invading cancer cells (examples indicated by arrows) were counted. The negative control for cytokeratin staining is shown in Supplementary Fig. 8g. **b** Effects of HGF/c-MET inhibition on cancer cell migration in vitro. Morphometric analysis demonstrated that exposure of matrices to HGF-neutralising antibody (Hi) and HGF-neutralising antibody + c-MET inhibitor (Hi + Ci) significantly inhibited cancer migration/invasion compared to matrices incubated with IgG or the c-MET inhibitor (Ci) alone. *p < 0.05 A vs. IgG; **p < 0.01 Hi + Ci vs. IgG; ^#^*p* < 0.05 Hi + G vs. Ci; *n* = 3 matrices per treatment, each containing a different human PSC preparation. **c** Representative cross-sections of matrices were stained for stem cell marker ALDH-1 (brown). The negative control for ALDH-1 staining is shown in Supplementary Fig. 8h. **d** Effect of HGF/c-MET inhibition on cancer stemness (ALDH-1 expression) in vitro. ALDH-1 staining demonstrated that HGF-neutralising antibody (Hi), c-MET inhibitor (Ci) and Hi+Ci significantly decrease stem cell marker, ALDH-1, expression in the tumours compared to IgG. *p < 0.05 Hi vs. IgG; *p < 0.05 Ci vs. IgG; **p < 0.01 Hi + Ci vs. IgG; *n* = 3 matrices per treatment, each containing a different human PSC preparation. **e** Effect of HGF/c-MET inhibition on cancer cell EMT in vitro. Vimentin staining demonstrated that Hi + Ci significantly decreased the expression of vimentin. **p < 0.01 Hi + Ci vs. IgG. E-cadherin staining revealed that c-MET inhibitor (Ci) and HGF-neutralising antibody + c-MET inhibitor (Hi + Ci) statistically significantly increased the expression of E-cadherin. *p < 0.05 Ci vs. IgG or Hi; **p < 0.01 Hi + Ci vs. IgG or Hi; *n* = 3 matrices per treatment, each containing a different human PSC preparation.

We found that while HGF or c-MET inhibition alone had variable effects on cancer migration, stemness and EMT, simultaneous inhibition of both the ligand and receptor (Hi + Ci) consistently reduced cancer cell migration/invasion into collagenous matrices, stem cell marker expression and EMT (Fig. 5a–e).

## Discussion

In this study, we have demonstrated for the first time that inhibition of both the ligand and the receptor of the HGF/c-MET pathway (using a neutralising antibody against HGF and a small-molecule c-MET receptor inhibitor) in combination with a chemotherapeutic agent (gemcitabine), that is, (triple therapy, Hi + Ci + G) significantly reduced tumour size and, more importantly, completely eradicated metastasis in a clinically relevant *advanced* pancreatic cancer model.

Our pancreatic cancer treatment strategy included chemotherapy and HGF/c-MET inhibition. The reason for choosing the latter is that it is one of the signalling pathways mediating the interactions between stromal and cancer cells and plays a major role in promoting cancer progression. We have previously demonstrated that PSCs produce HGF, but do not have its receptor.^16^ On the other hand, both pancreatic cancer cells and endothelial cells express the HGF receptor, c-MET.^13,16^ It is well established that cancer cells secrete growth factors (i.e. PDGF, VEGF, TGF-β, FGF-2 and etc.), which favours recruitment of different types of stromal cells, including PSCs, endothelial cells and immune cells to create a pro-tumour microenvironment.^18^ Cancer cells induce PSC activation through various growth factors listed above and PSCs produce HGF, which acts on cancer cells, thus forming a feed-forward loop. HGF also stimulates angiogenesis, further supporting tumour growth.^13^ Therefore, blocking the entire HGF/c-MET pathway represents a promising treatment approach for pancreatic cancer.

After 6 weeks of treatment, mice treated with gemcitabine, either as a single agent or in dual combination with HGF or c-MET inhibition, exhibited smaller tumours than IgG-treated controls, but statistically significant differences were only observed in G + Ci and the triple therapy (Hi + Ci + G) groups. Since reductions in tumour size could be due to a decrease in cancer cell number and/or fibrosis in tumours, these parameters were assessed histologically. Immunohistochemistry for the cancer cell marker cytokeratin, the proliferation marker PCNA and the apoptosis marker TUNEL, revealed that HGF/c-MET inhibition per se had no effect on cancer cell number. However, significantly decreased cancer cell numbers (due to decreased proliferation as well as increased apoptosis) were observed in tumours treated with gemcitabine alone or in dual or triple combinations with HGF/c-MET inhibitors.

With regard to fibrosis, collagen deposition was largely unaffected by the treatments, except interestingly, the HGF-neutralising antibody + c-MET inhibitor (Hi + Ci)-treated tumours, which showed a significant decrease in collagen content when compared with those treated with gemcitabine alone. The reason for this decrease is unclear since PSC activation (as assessed by α-SMA staining) in this group was unchanged compared to other groups. Overall, the reduction in tumour size appears to be due more to the decrease in cell density rather than any change in fibrosis. Furthermore, the loss of cancer cells is likely due to cytotoxicity of gemcitabine killing the chemo-sensitive cancer cell populations in the tumours, since significant decreases in cancer cell numbers (and tumour volume) were mainly observed in all groups treated with gemcitabine.

Our in vitro results support the above in vivo findings. Studies with our 3D organotypic cultures (involving the seeding of cancer cells on top of PSC-containing collagen matrices) showed that inhibition of HGF and c-MET (either alone or in combination) did not alter cancer cell proliferation. However, gemcitabine alone, or in combination with HGF/c-MET inhibition, significantly reduced proliferation. Treatment with HGF-neutralising antibody + c-MET inhibitor (Hi + Ci), as well as gemcitabine in combination with HGF/c-MET inhibition (Hi + Ci + G) significantly increased apoptosis of cancer cells.

With regard to metastatic spread, HGF/c-MET inhibitors as single or dual agents failed to show any efficacy, an effect similar to the lack of efficacy of these compounds on tumour volume. The inhibition of tumour growth by gemcitabine alone failed to translate into statistically significant inhibition of metastasis. However, gemcitabine in combination with HGF or c-MET inhibitions did significantly reduce metastatic burden. Most notably, triple therapy (Hi + Ci + G) completely eliminated metastasis, even at the advanced stage of pancreatic cancer modelled in our orthotopic study.

To determine whether cancer cell invasion/migration was affected by the different treatments in vitro, we conducted studies using our 3D cultures. We were unable to test treatments containing gemcitabine in the 3D set-up, because prolonged incubation with gemcitabine (necessary for invasion studies through collagen matrices), resulted in the death of all tumour cells on the top of the 3D matrix. However, we were able to assess the effects of HGF/c-MET inhibition on cancer cell invasion. Surprisingly, and in contrast to the lack of effect on metastasis in vivo, we found that inhibition of both HGF and c-MET (over a period of 4 weeks in culture) significantly decreased the invasion of cancer cells into the collagen matrices.

The reasons for this apparent discrepancy between our in vivo findings (no anti-metastatic effect of HGF + c-MET inhibition) and the significant anti-invasive effect in vitro, are not entirely clear. One possible explanation may be that by the time the treatments are instituted in vivo, the cancer cells have already disseminated and HGF/c-MET inhibition by itself cannot exert any evident anti-cancer effects. However, based on our published work with the early model of pancreatic cancer,^11^ and on the current in vitro data, it is likely that dual inhibition of HGF and c-MET can block/prevent the occurrence of new metastases, but has little effect on established metastasis.

Metastasis is driven by two major factors—EMT and increased survival/stemness of cancer cells. In tumours of mice treated with gemcitabine alone, expression of the stem cell marker DCLK1 was elevated (although not reaching statistical significance). This effect is similar to that observed in our published study with early pancreatic cancer.^11^ DCLK1 expression was unchanged in all other treatment groups, with the exception of the triple therapy group, where it was significantly reduced compared to the gemcitabine-alone group. Our in vitro data with the 3D model indicated that HGF + c-MET inhibition significantly reduces cancer cell stemness (as assessed by ALDH-1 expression). Thus, the absence of metastasis in the triple therapy group may be partially due to the loss of cancer cells (secondary to the cytotoxic effect of gemcitabine) and the decreased stemness of the surviving cells due to HGF/c-MET inhibition.

With regard to EMT, in tumours of mice treated with HGF antibody + c-MET inhibitor (Hi + Ci), expression of E-cadherin was increased, while the expression of vimentin was reduced indicating inhibition of EMT. With the remaining groups, changes in the expression of E-cadherin and vimentin were not significant, although trends to increased vimentin expression were observed in all mice receiving gemcitabine. Our in vitro 3D culture studies supported our in vivo observations for HGF + c-MET inhibition on EMT, with cancer cells expressing a significant increase in E-cadherin and a significant decrease in vimentin in this treatment group. Thus, the observed absence of metastasis in the triple therapy group may also reflect the important contribution of the HGF/c-MET inhibition component of the triple therapy, which significantly downregulates EMT of cancer cells.

We recognise that our pre-clinical model is limited by the use of only one cancer cell line to produce pancreatic tumours and by its immunocompromised background. We selected AsPC-1 as the cell line for our studies, because this is an aggressive metastatic cell line that allows simulation of the most common scenario in the clinic, that is, a tumour that has already metastasised at the time of diagnosis. Other cell lines such as MiaPaCa-2, Panc-1 and BxPC3 have been used in studies published by us and others^5,6^ to demonstrate that the presence of PSCs accelerates cancer progression in a manner similar to that with AsPC-1 (thus the orthotopic model itself would not be different with different cell lines). Moreover, the majority of commercially available cell lines exhibit high expression of c-MET and hence the response of orthotopic tumours produced by other cell lines + PSCs, would be reasonably expected to be similar to our findings with the current model. Most importantly, a noteworthy feature of our model is that we have used primary PSC preparations from several different patients to account for the now well-recognised heterogeneity of PSCs within cancer stroma. We believe that this is a distinct advantage of our model, compared to those produced with different cancer cell lines but the same fibroblast/mesenchymal cell line.

With regard to the immune status of the model, a major advantage of using an immunocompromised model in the current study is that we have been able to demonstrate specific effects of HGF inhibition and c-MET inhibition on ***human*** cancer cells and human PSCs, thereby increasing the translational potential of the findings. Interestingly, a recent paper by Giannoni et al.^19^ has reported that in chronic lymphocytic leukaemia, HGF secreted by mesenchymal cells not only stimulates leukaemic cell proliferation but also drives monocytes/macrophages to an M2, immune suppressive phenotype. Thus, it may be expected that in an immunocompetent setting our tested approach of HGF/c-MET inhibition would be even more effective since it will not only block cancer cell growth due to PSC–cancer cell interactions but will also inhibit immune suppression, thereby allowing T cell-mediated cytotoxic effects on cancer cells.

The HGF/c-MET pathway has been targeted in clinical studies in gastro-oesophageal, ovarian and primary peritoneal cancers, with largely negative results. However, it must be noted that these studies only involved inhibition of either the ligand or the receptor of this pathway with or without chemotherapy. Our findings clearly indicate that it is the triple therapy (the combination of a chemotherapeutic agent with compounds that inhibit both arms of HGF/c-MET pathway) that gives the best outcomes in terms of preventing cancer progression.

**Fig. 6:**
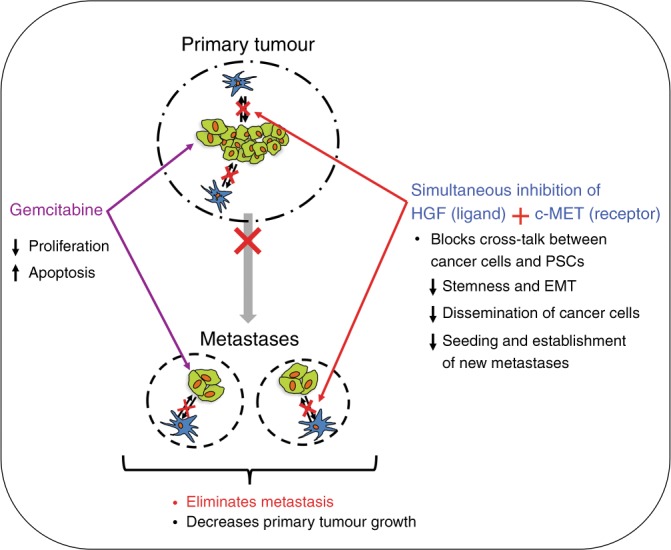
This figure summarises the postulated mechanisms underpinning the effects of triple therapy on advanced pancreatic cancer. Gemcitabine causes cancer cell death in both the established tumours and metastases via increased apoptosis and decreased proliferation of cancer cells. At the same time, inhibition of both the ligand and receptor components of the HGF/c-MET pathway blocks PSC–cancer cell interactions, thereby inhibiting EMT and stemness. These effects inhibit dissemination of cancer cells from the primary tumour and also oppose establishment of new metastases. This two-pronged approach targeting cancer cells combined with effective inhibition of a major pathway that mediates stromal–tumour interactions eliminates metastasis and decreases tumour growth, even at an advanced stage of pancreatic cancer.

We postulate that the following factors underpin the observed effects of the triple therapy regimen as illustrated in Fig. 6:

1. At a stage where the primary tumour and metastases are well established (4 weeks after orthotopic implantation), gemcitabine destroys chemo-sensitive cancer cells in the pancreas and in metastatic nodules.
2. HGF produced by PSCs within the primary tumour is neutralised by the neutralising antibody, depriving its receptor c-MET (on cancer cells and endothelial cells), of ligand binding. Similar effects occur at metastatic sites, since we have previously demonstrated that PSCs from primary tumours are also found within metastatic nodules.^7^
3. In addition to the lack of a ligand, activation of c-MET on cancer cells and endothelial cells in both primary tumour and metastatic nodules is prevented by the c-MET inhibitor. This c-MET receptor blockade may also inhibit the known cross-talk of c-MET with other receptors such as EGFR.^20^
4. Downstream signalling pathways (such as MAPK and PI3K), which regulate HGF/c-MET-induced effects on cancer cells and endothelial cells,^11,13,16^ are not activated, preventing cell proliferation and migration/invasion, and reducing growth factor and cytokine secretion (which in turn interrupts further activation of PSCs).

Taken together, the above effects inhibit the growth of primary tumour, eliminate existing metastases completely and, importantly, prevent the formation of new metastases, by inhibiting EMT and stemness and by removing the pro-tumorigenic interactions of PSCs and cancer cells seeded at distant sites.

In summary, we submit that the advanced model of orthotopic pancreatic cancer used in our study represents a scenario commonly seen in the clinic, where a majority of patients present with locally advanced and/or metastatic disease at the time of diagnosis. Our findings indicate that targeting of both the ligand and receptor arms of the HGF/c-MET pathway combined with a chemotherapeutic agent could be a highly effective approach clinically in the neoadjuvant setting, enabling downsizing of tumour and elimination of micro-metastases and consequently improving the suitability of the patient for surgical resection.

## Supplementary information

Supplementary text clean ver and supplementary figures

## Data Availability

All data and materials are published in the manuscript, supplementary materials on journal website or available on request.
