# Supplementary material for: Targeting the HGF/c-MET pathway in advanced pancreatic cancer: a key element of treatment that limits primary tumour growth and eliminates metastasis
Source: Br J Cancer. 2020 Mar 23;122(10):1486–95. doi: 10.1038/s41416-020-0782-1 (PMC7217847; doi:10.1038/s41416-020-0782-1)

SUPPLEMENTARY METHODS

Materials and methods

Reagents

The reagents included Iscove's Modified Dulbecco's Medium, RPMI Medium 1640, fetal bovine serum, glutamine, penicillin and streptomycin (for cell culture) from Invitrogen; Hyclone SF4MAb Medium from Thermo Fisher Scientific; anti-α-smooth muscle actin (αSMA) antibody, Soybean oil from Sigma; anti-glial fibrillary acidic protein (GFAP) antibody, anti-cytokeratin antibody, anti-proliferating cell nuclear antigen (PCNA) antibody and 3,3'-diaminobenzidine (DAB) tetrahydrochloride substrate from DAKO; anti-CD31 antibody, anti-Ki-67 antibody from Abcam; Safil 5/0 Polyglycolic acid absorbable surgical suture from B.Braun; Ketamine, Xylazine, isoflurane and Flunixin from Cenvet Australia; Isotype IgG, HGF inhibitor, Rilotumumab/AMG102 and Compound A from Amgen Inc.; Gemcitabine from McFarlane Medical & Scientific Australia; PHA-665752, from Tocris Bioscience.

Orthotopic model of advanced pancreatic cancer

Animal specifics

8-10 week old female athymic mice (spontaneous mutation *Foxn1*^{nu}, also named as Balb/c nude mice) were used. The body weight range of the mice was 17-21 grams in this study.

Animal housing

Mice were housed in the Biological Resources Centre of Ingham Institute. This facility is certified to OGTR Physical Containment level 2 (PC2), as an accredited Animal Research Establishment by the Department of Primary Industries of Australia. The

caging system was Allentown Individually Ventilated Caging and Racks with HEPA supply and exhaust blowers. Each cage was provided with Aspen Bedding, Crink-L nesting material, wooden chew stick and red mouse igloo as environment enrichment. Irradiated Mouse Breeder Pellets and Reverse Osmosis and acidified water were given ad libitum.

Details of drugs administered in this study:

1. Anaesthesia: Ketamine 80 mg/kg + Xylazine 10 mg/kg in 200μl water for injection was administered through intraperitoneal injection (IP). This combination of anaesthetics was selected for optimal short-term anaesthesia, based on our experience with this anaesthetic regimen over the past 15 years. The IP injection of anaesthetics was performed in Class II Biosafety cabinet 5 minutes prior surgery. The surgical procedures were usually scheduled in the afternoon of a given day.
2. Analgesia: Flunixin 2.5mg/kg was administered subcutaneously at the nape of mouse upon completion of surgery. Buprenorphine 0.05 mg/kg was given subcutaneously to relieve the mice from pain or distress as detailed in the "monitoring" section below.
3. Treatment groups: The treatment was initiated 4 weeks after surgical implantation of cells.
   i) IgG group:

      a) Isotype IgG served as control for HGF neutralising antibody. It was administered through IP in a dose of 300 μg in 200 μl sterile PBS to each mouse, twice weekly.

b) Soybean oil (served as solvent/vehicle control for c-MET inhibitor) 10 ml/kg (10 µl/g) was administered through oral gavage daily.

ii) G group:

Gemcitabine 75 mg/kg in 100 µl saline was administered through IP in 100 µl saline for injection, twice weekly. This dosage regimen was determined by our previous dose response studies for efficacy and tolerance in the same strain of mice. It has been shown to be well tolerated by the mice (1, 2).

iii) Hi group of mice:

HGF inhibitor, Rilotumumab/AMG102, is a human HGF neutralising antibody from Amgen Inc. Thousand Oaks, CA, USA. This HGF neutralising antibody 300 µg per mouse was administered through IP in 200 µL sterile PBS, twice weekly. This dosage regimen was determined by our previous dose response studies for efficacy and tolerance in the same strain of mice. It has been shown to be well tolerated by the mice (1, 2).

iv) Ci group:

c-MET inhibitor, Compound-A, is a small molecule inhibitor that prevents the activation of c-MET (HGF receptor) manufactured by Amgen Inc. This c-MET inhibitor was dissolved at 60 mg per 10 ml of soybean oil and given at 10 ml/kg (10 µl/g) through oral gavage daily. The dosage of c-MET inhibitor was 60 mg/kg. This dosage regimen was recommended by the manufacturer based upon their studies of this compound. It has been shown to be well tolerated by the mice (2).

v) Hi+Ci group:

a) HGF neutralising antibody 300 µg per mouse was administered through IP in 200 µL sterile PBS, twice weekly.

b) c-MET inhibitor 60 mg/kg was administered through oral gavage daily.

vi) Hi+G group:

a) HGF neutralising antibody 300 μg per mouse was administered through IP in 200 μL sterile PBS, twice weekly.

b) Gemcitabine 75 mg/kg was administered through IP in 100 μl saline for injection, twice weekly.

vii) Ci+G group:

a) c-MET inhibitor 60 mg/kg was administered through oral gavage daily.

b) Gemcitabine 75 mg/kg was administered through IP in 100 μl saline for injection, twice weekly.

viii) Hi+Ci+G group:

a) HGF neutralising antibody 300 μg per mouse was administered through IP in 200 μL sterile PBS, twice weekly.

b) c-MET inhibitor 60 mg/kg was administered through oral gavage daily.

c) Gemcitabine 75 mg/kg was administered through IP in 100 μl saline for injection, twice weekly.

The combinations of Hi+Ci, Hi+G, Ci+G and Hi+Ci+G have been assessed in our previous tolerance studies in the same strain of mice. They have been shown to be well tolerated by the mice (2).

Method of euthanasia: CO_2 asphyxiation and exsanguination. The mouse was individually placed into a transparent chamber with a CO_2 inlet. Normal air in the chamber was gradually displaced by CO_2 by adjusting the flow rate using a regulator.

Once the mouse was comatose. It was moved to procedure table. A cardiac puncture was performed immediately to exsanguinate animal.

Experimental procedures

Mice were acclimatised and monitored for one week before surgery and subjected to the following procedures.

Anaesthesia

Mice were anaesthetised with 80 mg/kg Ketamine + 10 mg/kg Xylazine through IP (volume of injectate 200µl) in a Class II biosafety cabinet before the surgery and moved into a separate cage (same as its holding cage) until adequate anaesthesia was achieved. Depth of anaesthesia was assessed by toe pinch reflex. In case of inadequate depth of anaesthesia, a further dose of 20mg/kg of Ketamine was administered IP. Mice were monitored continuously during the operative procedure for respiration, cyanosis and reflexes. This anaesthetic method has been established in our lab for the past 15 years. It has been demonstrated to be safe and effective in over 200 Balb/c nude mice in our previous studies (1-4) which had been approved by Animal Care and Ethics Committee of the University of New South Wales, Sydney, Australia.

Surgery

After adequate anaesthesia was achieved, the mouse was moved onto a small animal surgery pad that was covered with a sterile drape (keeping the mouse warmed during the procedure in a Class II biosafety cabinet). The abdomen was prepared for surgery using Betadine-soaked gauze and 70% ethanol wipes. A left lateral subcostal incision was made in the abdominal wall to exteriorise the body and tail of the pancreas and cell

implantation was performed. Pancreas was then gently returned into the abdominal cavity. The incision was closed with absorbable synthetic suture and skin clips for small animal surgery. Flunixin was administered subcutaneously into the nape of the mouse at a dose of 2.5mg/kg dose in saline. Skin clips were removed 7 days post surgery.

Monitoring

Animals were continuously monitored during surgery and for the first 4 hours after recovery from anaesthesia. This was followed by daily monitoring for the duration of the experiments. If the animal was assessed to have pain during handling for weighing, inspection of the incision or abdominal palpation, Buprenorphine was administered at a dose of 0.05 mg/kg subcutaneously. In the event of untreatable distress (hunched posture, lack of movement, lack of grooming, abnormal feeding patterns, jaundice, abdominal distension, vocalization), or poor body condition score, or in the event of loss of more than or equal to 20% body weight, animals were humanely euthanised by CO_2 asphyxiation and exsanguination (see this supplementary materials page 4 paragraph 3 for details of euthanasia).

Bioluminescent imaging

The IVIS Lumina II imaging system was used. Mice were injected intraperitoneally with D-Luciferin (150mg/kg) in a Class II Biological Safety Cabinet located immediately adjacent to the IVIS Lumina II imaging chamber. After IP injection, mice were moved into a separate cage (the same as its holding cage) until imaging was due in 5 minutes. Anaesthesia was induced in the animals using an isoflurane vapourising chamber. After adequate anaesthesia was achieved, mice were then transferred to the anaesthetic/imaging chamber of the IVIS system - this has a volatile anaesthetic

delivery system which allows maintenance of anaesthesia depth during the imaging process (similar to a nosecone system). Mice were monitored for depth of anaesthesia by toe-pinch reflex. At the end of the imaging procedure, mice were returned to their housing cages which had been placed on heating pads. The animals were then monitored for 30 minutes until awake and alert before being returned to the cage rack.

Treatment group allocation, sample size and power calculation

Eight treatment groups were designed in this study. Based on our 15 years' experience and previously published studies with this orthotopic mouse model of pancreatic cancer, n = 6 mice per group is calculated to allow detection of a 30% difference between groups with 80% power at an alpha of 5%. In total, 48 mice were included in this study.

Four weeks after surgery, the size of primary tumours was similar as assessed by bioluminescent imaging (Results Figure 1A). Therefore, mice random allocation was used to assign mice to the different treatment groups.

The treatment groups and regimen are detailed in the Materials and Methods section of the main text in the manuscript. Among different treatments in this study, first, Soybean oil (solvent/vehicle control) or c-MET inhibitor was administered to corresponding groups via oral gavage every day. After one hour, isotype IgG or HGF neutralising antibody were administered to corresponding groups via IP injection on the date when this treatment was due (twice weekly). After another 45 minutes, gemcitabine was administered to corresponding groups via IP injection when this drug was due (also twice weekly). Treatment was delivered in a random sequence for the mice of the same treatment group. Mice were humanely euthanised by CO_2 asphyxiation and

exsanguination after 6 weeks of treatment (See this supplementary materials page 4 paragraph 3 for details of euthanasia).

Rationale of using mice for this study

This study examined the effects of growth factor inhibitors plus chemotherapy on advanced pancreatic cancer progression. The nude mouse orthotopic xenograft model is a physiologically relevant model for this study because it allows the determination of tumour growth and behaviour in a functionally intact system. This allows the study of the tumour in the context of the interplay between important tissue components - endocrine (hormones), autocrine (molecules secreted by cells themselves) and paracrine (molecules secreted by one cell that affect the function of a neighbouring cell). Since such conditions could not be simulated in a culture dish or organoids, it was essential to use intact animals for this study.

SUPPLEMENTARY DATA

1. PSC activation in an advanced pancreatic cancer model

   PSC activation was evaluated using immunohistochemistry staining for the PSC activation marker αSMA. The results showed that PSC activation was not influenced by the treatments (Results expressed as Integrated Optical Density Units - IgG: 171.7±1.5, G: 156±1.5, Hi: 152.3±2.2, Ci: 144.9±2.1, Hi+Ci: 164.3±2.2, Hi+G: 161.3±1.3, Ci+G: 158±2.1; Hi+Ci+G: 161.2±1.6).

SUPPLEMENTARY FIGURE LEGENDS

Supplementary Figure 1

Effects of HGF/c-MET inhibition ± gemcitabine on collagen deposition in primary tumours.

A: Representative Sirius Red-stained images. Scale bars = 50 μm.

B: Gemcitabine alone elevated tumour collagen deposition, but this did not achieve statistical significance. Treatment with HGF neutralising antibody + c-MET inhibitor (Hi+Ci) significantly reduced collagen deposition compared to the gemcitabine (G) group. **p < 0.01, Hi+Ci *vs* G, n = 6 mice/group.

Supplementary Figure 2

Effects of HGF/c-MET inhibition ± gemcitabine on cancer cell numbers.

A: Representative photomicrographs of immunostaining for cytokeratin (cancer cell marker indicating cancer cell density in each group). Scale bars = 50 μm. The negative control for cytokeratin staining (sections stained with equivalent concentration of isotype IgG) is shown in Supplementary Figure 8j.

B: The number of cytokeratin positive cells (cancer cells) per high power field (HPF) was significantly lower in tumours from mice treated with gemcitabine (G), HGF neutralising antibody + gemcitabine (Hi+G), c-MET inhibitor + gemcitabine (Ci+G) and triple therapy (Hi+Ci+G), compared with IgG, Hi, Ci or Hi+Ci treated mice. ***p < 0.001, G, Hi+G, Ci+G or Hi+Ci+G *vs* IgG, Hi, Ci or Hi+Ci; n = 6 mice/group.

Supplementary Figure 3

A: Representative photomicrographs of immunostaining for Ki-67 (cell proliferation marker). Scale bars = 50 µm. The negative control for Ki-67 staining is shown in Supplementary Figure 8k.

B: Effects of HGF/c-MET inhibition ± gemcitabine on cancer cell proliferation. Treatment with c-MET inhibitor + gemcitabine (Ci+G) or triple therapy (Hi+Ci+G) decreased the number of Ki-67 positive cells (proliferating cells) per high power field in the tumours. #p = 0.092, Ci+G *vs* IgG; $^{\$}$p = 0.068, Hi+Ci+G *vs* IgG; n = 6 mice/group.

Supplementary Figure 4

A: Representative photomicrographs of all treatment groups immunostained for vimentin (dark brown). Scale bars = 50 µm. The negative control for vimentin staining is shown in Supplementary Figure 8d.

B: Representative photomicrographs of immunostaining for E-cadherin (brown cell membrane staining) of all treatment groups. Scale bars = 50 µm. Scale bars = 50 µm. The negative control for E-cadherin staining is shown in Supplementary Figure 8e.

Tumours from HGF neutralising antibody + c-MET inhibitor treated mice (Hi+Ci) demonstrated highest E-cadherin and lowest vimentin expression compared to the rest of the groups, indicating an inhibition of EMT. The highest vimentin expression among all groups was observed in tumours from mice treated with gemcitabine alone (G). n = 6 mice/group.

Supplementary Figure 5

Representative photomicrographs of immunostaining for DCLK1 (stem cell marker) of all treatment groups. Triple therapy (Hi+Ci+G) significantly reduced DCLK1 expression in tumours compared to that in the gemcitabine (G) and c-MET inhibitor (Ci) treated groups *$p < 0.05$, n = 6 mice/group. Scale bars = 50 µm. Scale bars = 50 µm. The negative control for DCLK1 staining is shown in Supplementary Figure 8f.

Supplementary Figure 6

Effects of HGF/c-MET inhibition ± gemcitabine on cancer cell proliferation *in vitro*.

A: Representative photomicrographs of the 3D culture setting comprising collagen matrices containing PSCs with cancer cells (AsPC-1) seeded on the top of the matrices. These matrices were incubated with IgG, gemcitabine (G), HGF neutralising antibody (Hi), or c-MET inhibitor – PHA-665752 (Ci), Hi+Ci, Hi+G, Ci+G and Hi+Ci+G. Immunostaining for proliferating cell nuclear antigen (PCNA) was performed on paraffin sections of these matrices. PCNA positive cells were stained brown. The negative control for PCNA staining is shown in Supplementary Figure 8i.

B: The bar graph demonstrates a significant reduction in the number of proliferating cancer cells in matrices treated with gemcitabine (G) alone, as well as HGF neutralising antibody + gemcitabine (Hi+G), c-MET inhibitor + gemcitabine (Ci+G) and triple therapy (Hi+Ci+G) compared with matrices incubated with IgG, Hi, Ci or Hi+Ci. **$p < 0.01$, G, Hi+G, Ci+G or Hi+Ci+G *vs* IgG, Hi, Ci or Hi+Ci; n = 5 matrices per treatment, each containing a different human PSC preparation.

Supplementary Figure 7

HGF/c-MET inhibition ± gemcitabine on cancer apoptosis *in vitro*. TUNEL staining on sections of 3D matrices demonstrated that exposure to Hi+Ci, Hi+G, Ci+G and Hi+Ci+G statistically significantly increased cancer cell apoptosis, **$p < 0.01$, Hi+Ci, Hi+G, Ci+G or Hi+Ci+G *vs* IgG; #$p < 0.05$ Hi+Ci+G *vs* G, Hi, Ci or Hi+Ci; n = 5 matrices per treatment, each containing a different human PSC preparation.

Supplementary Figure 8

Relevant negative controls of immunostaining (sections stained with equivalent concentration of corresponding isotype IgG) as well as negative and positive controls of TUNEL staining. a: Negative control of immunostaining for PCNA; b: Negative control of TUNEL staining; c: Positive control of TUNEL staining; d: Negative control of immunostaining for vimentin; e: Negative control of immunostaining for E-cadherin; f: Negative control of immunostaining for DCLK1; g: Negative control of immunostaining for cytokeratin; h: Negative control of immunostaining for ALDH-1; i: Negative control of immunostaining for PCNA staining in 3D matrices; j: Negative control of immunostaining for cytokeratin; k: Negative control of immunostaining for Ki-67.

REFERENCES FOR SUPPLEMENTARY MATERIALS

1. Pothula SP, Xu Z, Goldstein D, Biankin AV, Pirola RC, Wilson JS, et al. Hepatocyte growth factor inhibition: a novel therapeutic approach in pancreatic cancer. Br J Cancer. 2016;114(3):269-80.
2. Pothula SP, Xu Z, Goldstein D, Merrett N, Pirola RC, Wilson JS, et al. Targeting the HGF/c-MET pathway: stromal remodelling in pancreatic cancer. Oncotarget. 2017;8(44):76722-39.
3. Vonlaufen A, Joshi S, Qu C, Phillips PA, Xu Z, Parker NR, et al. Pancreatic stellate cells: partners in crime with pancreatic cancer cells. Cancer Res. 2008;68(7):2085-93.
4. Xu Z, Vonlaufen A, Phillips PA, Fiala-Beer E, Zhang X, Yang L, et al. Role of pancreatic stellate cells in pancreatic cancer metastasis. Am J Pathol. 2010;177(5):2585-96.

Supplementary Fig 1

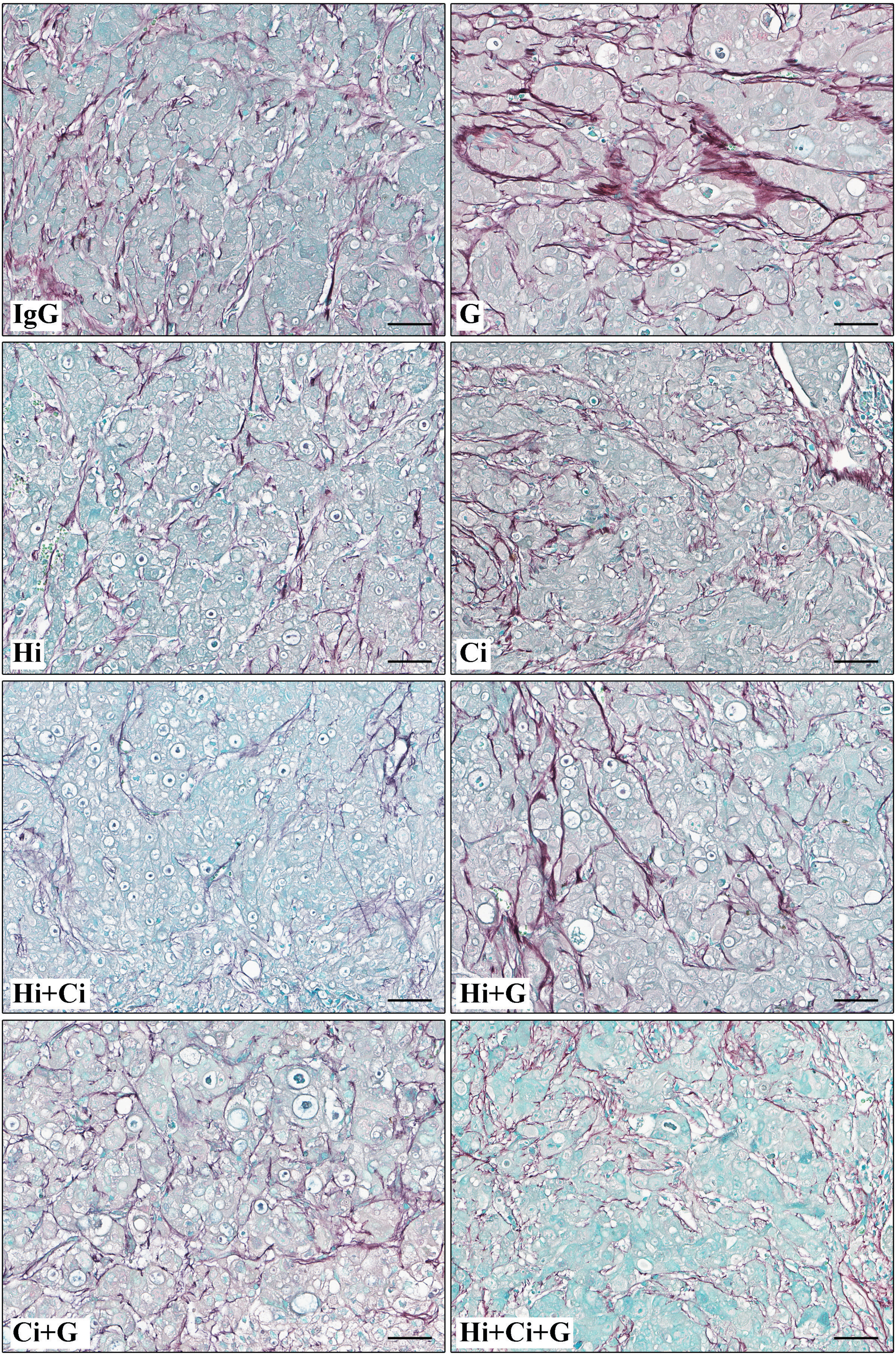

Supplementary Fig 1 B

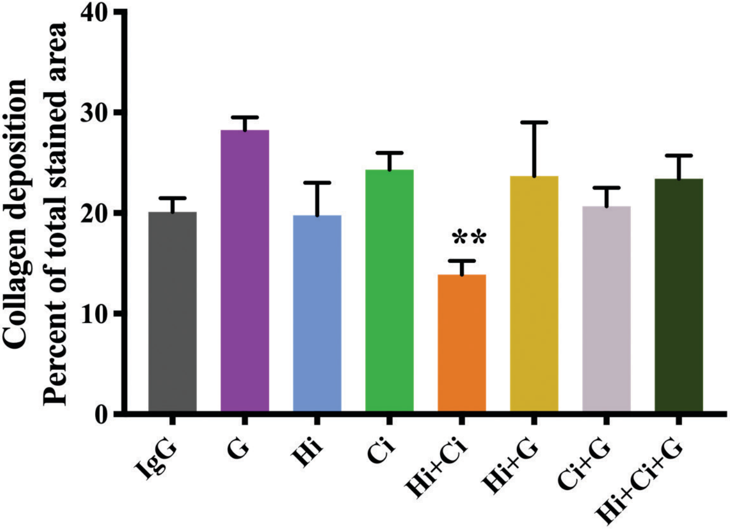

Supplementary Fig 2

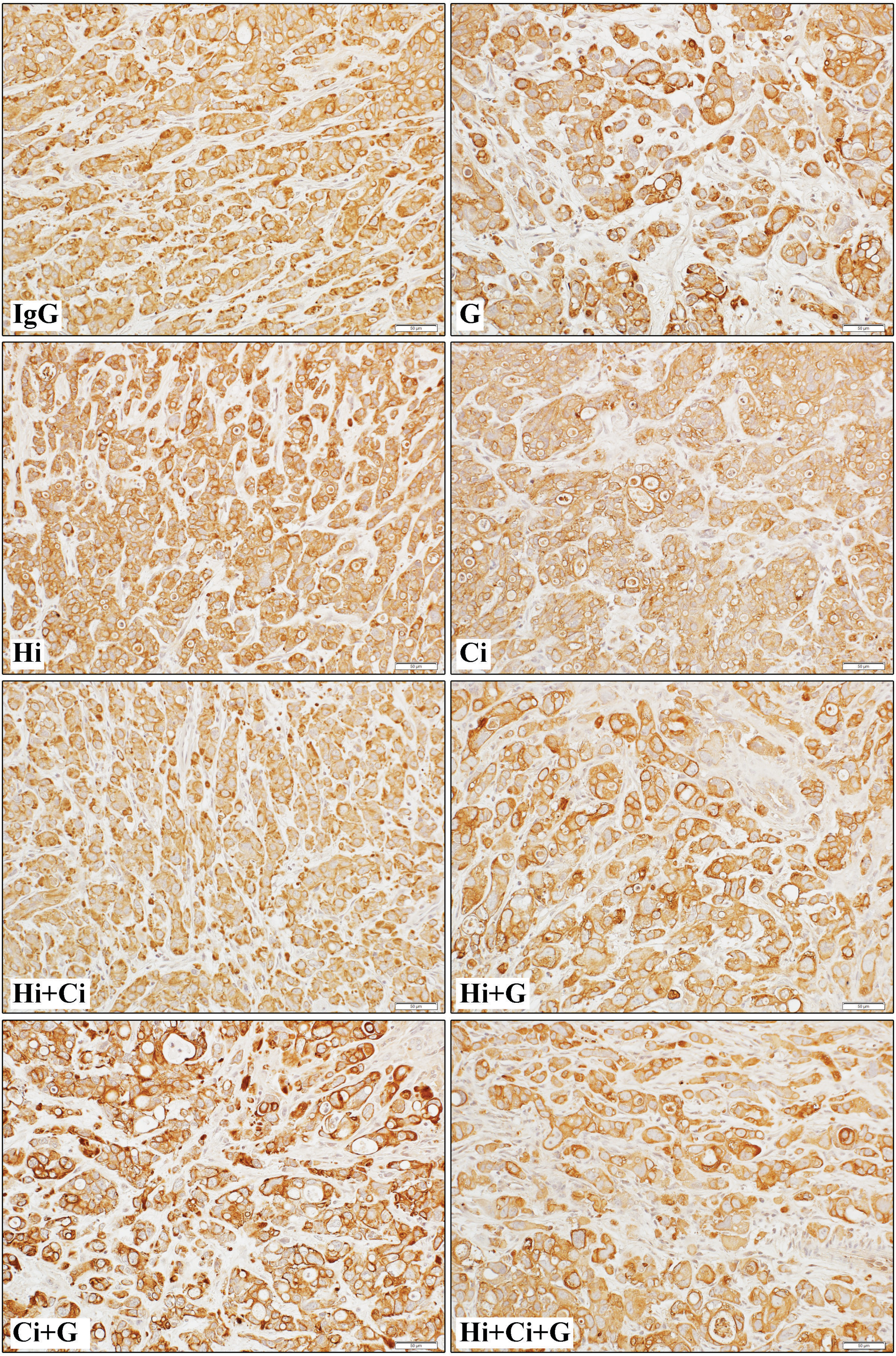

Supplementary Fig 2 B

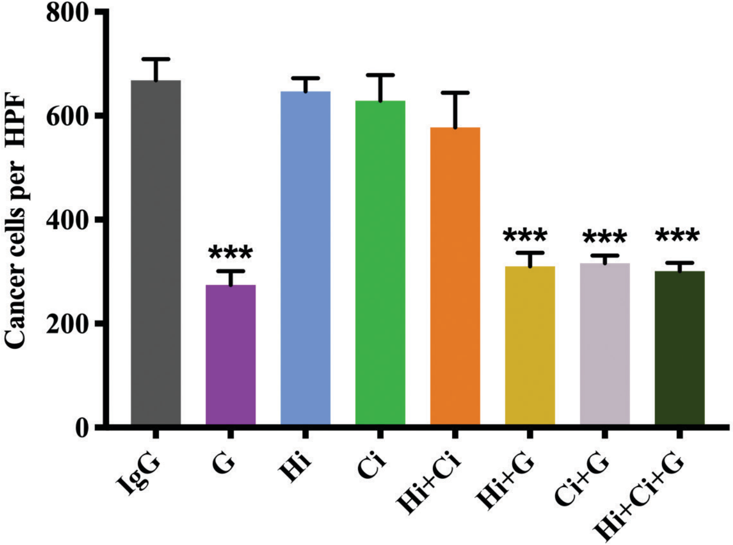

Supplementary Fig 3 A

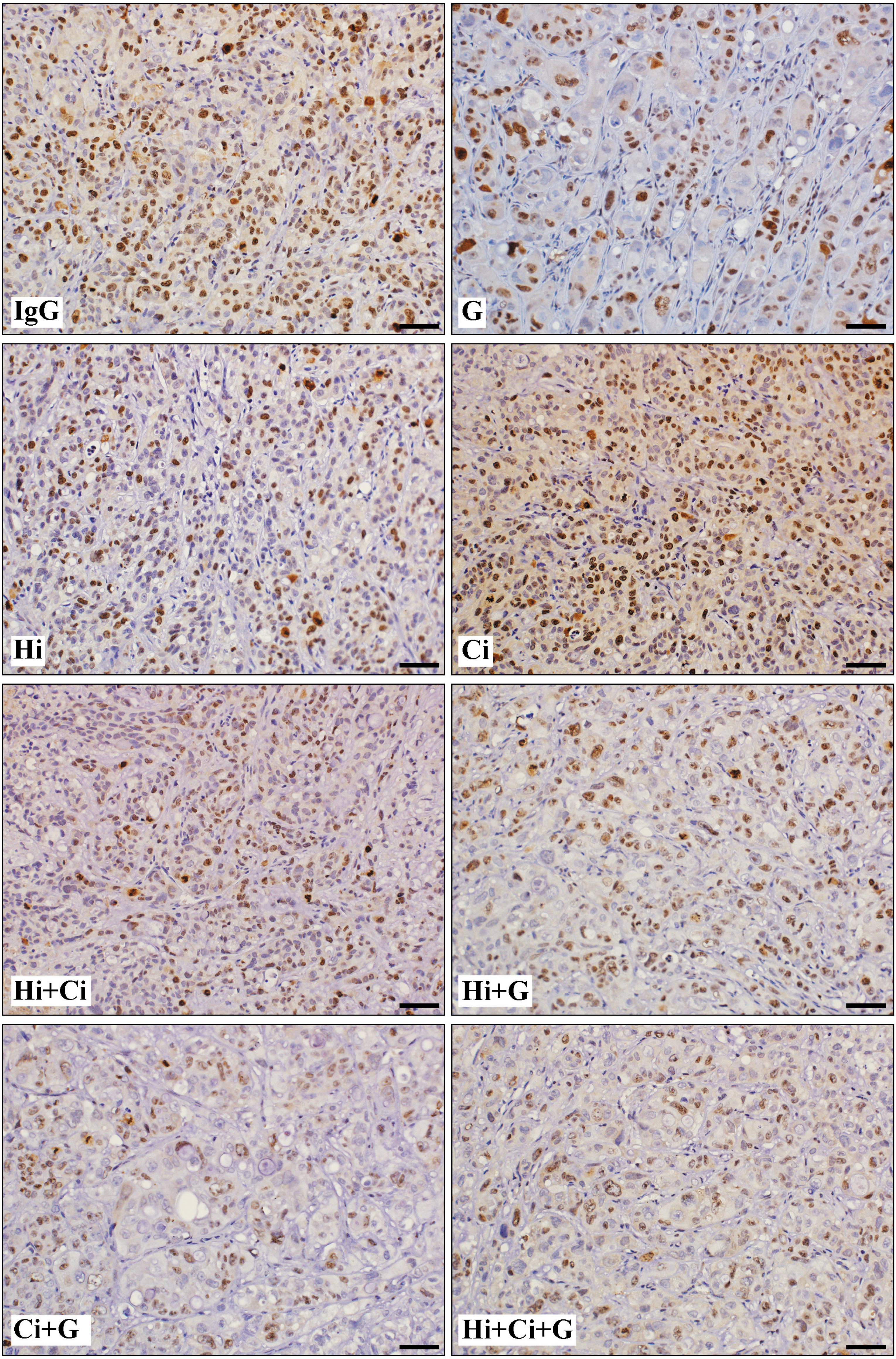

Supplementary Fig 3 B

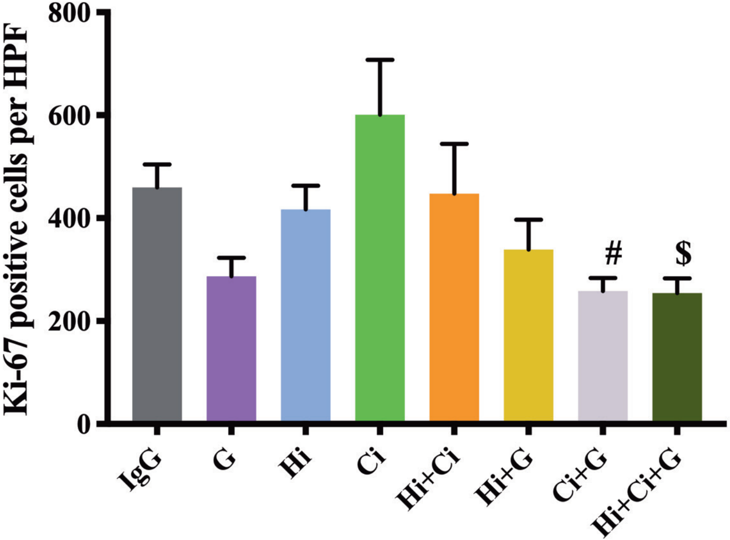

Supplementary Fig 4 A

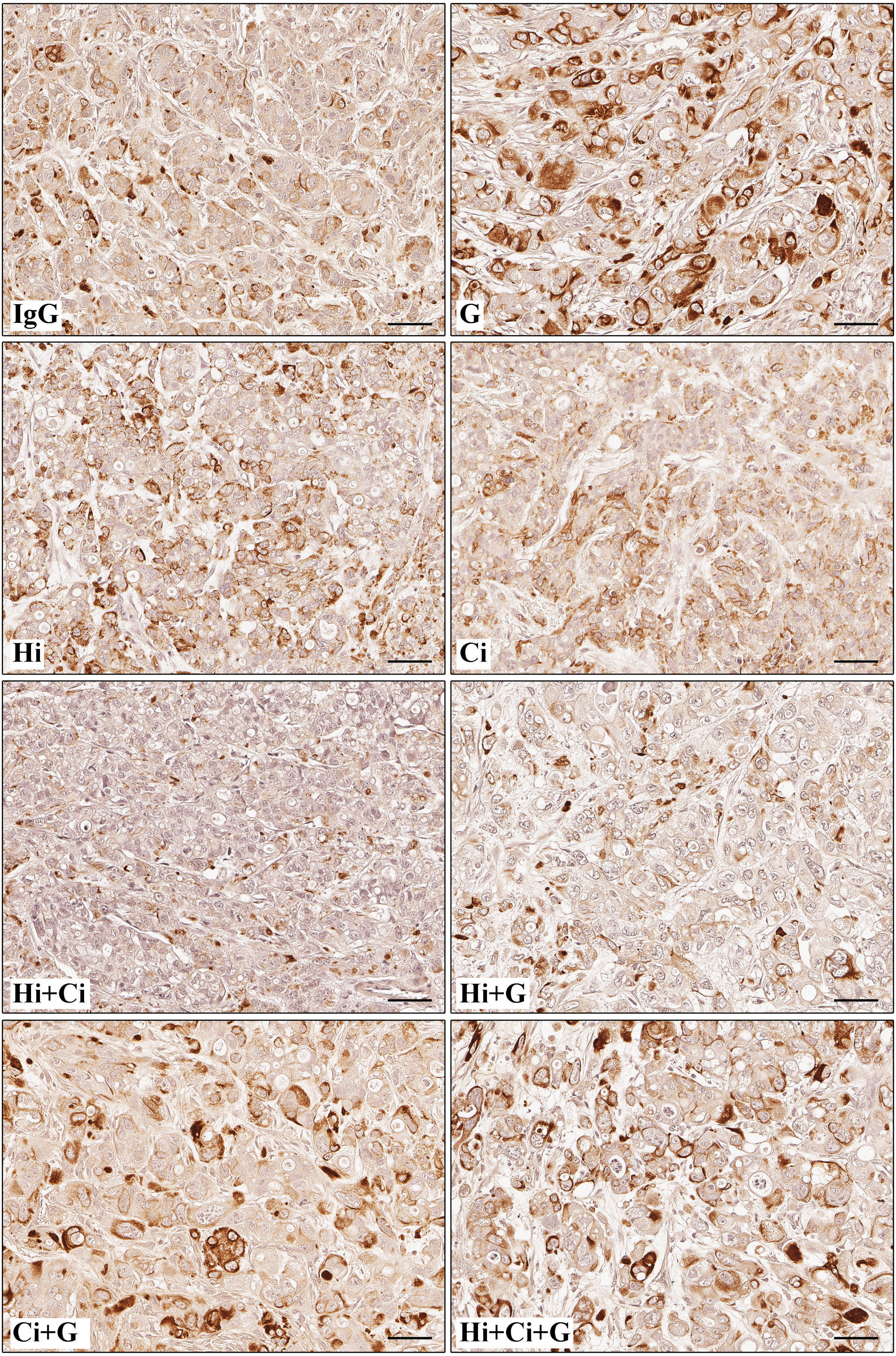

Supplementary Fig 4 B

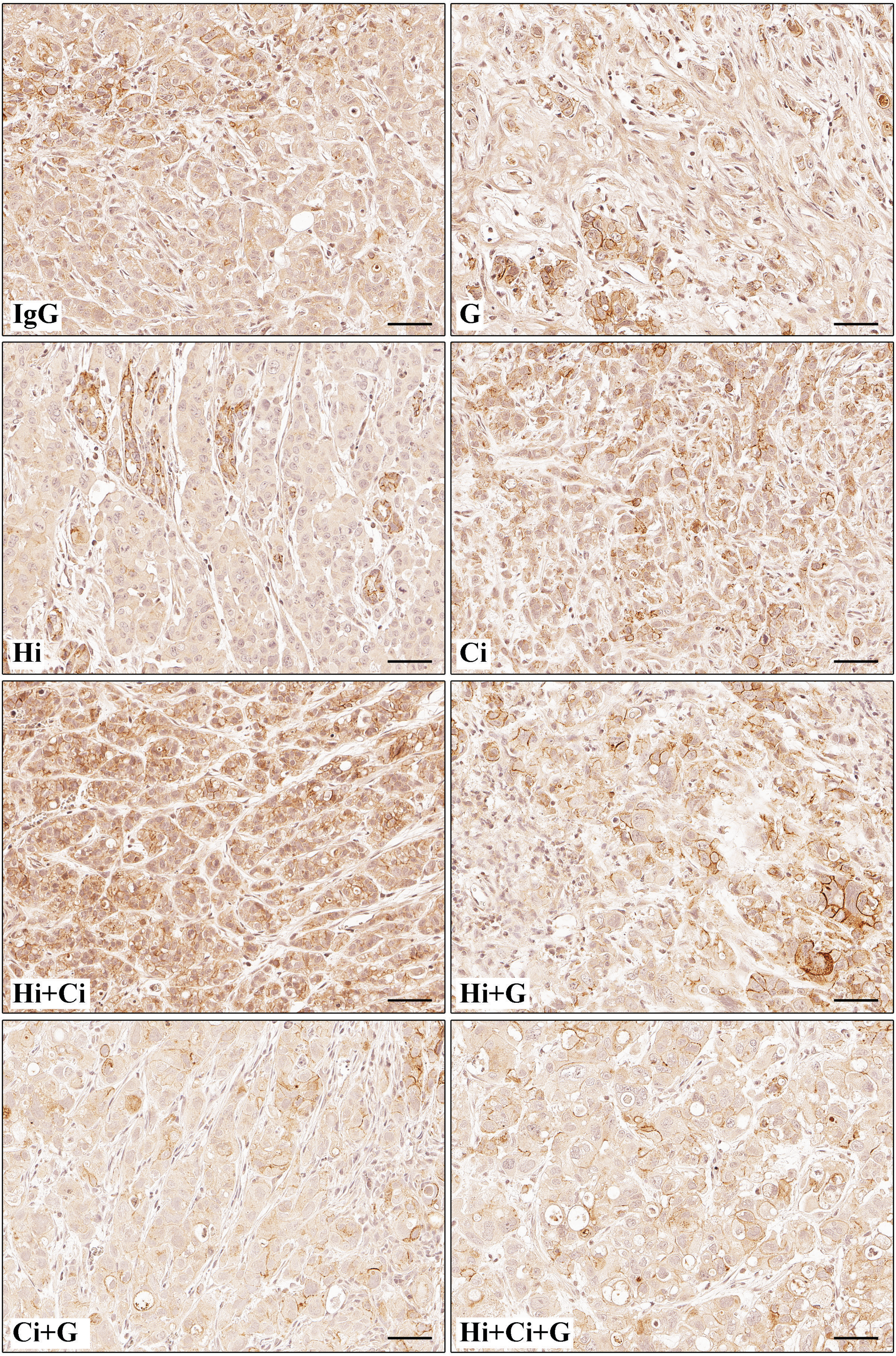

Supplementary Fig 5

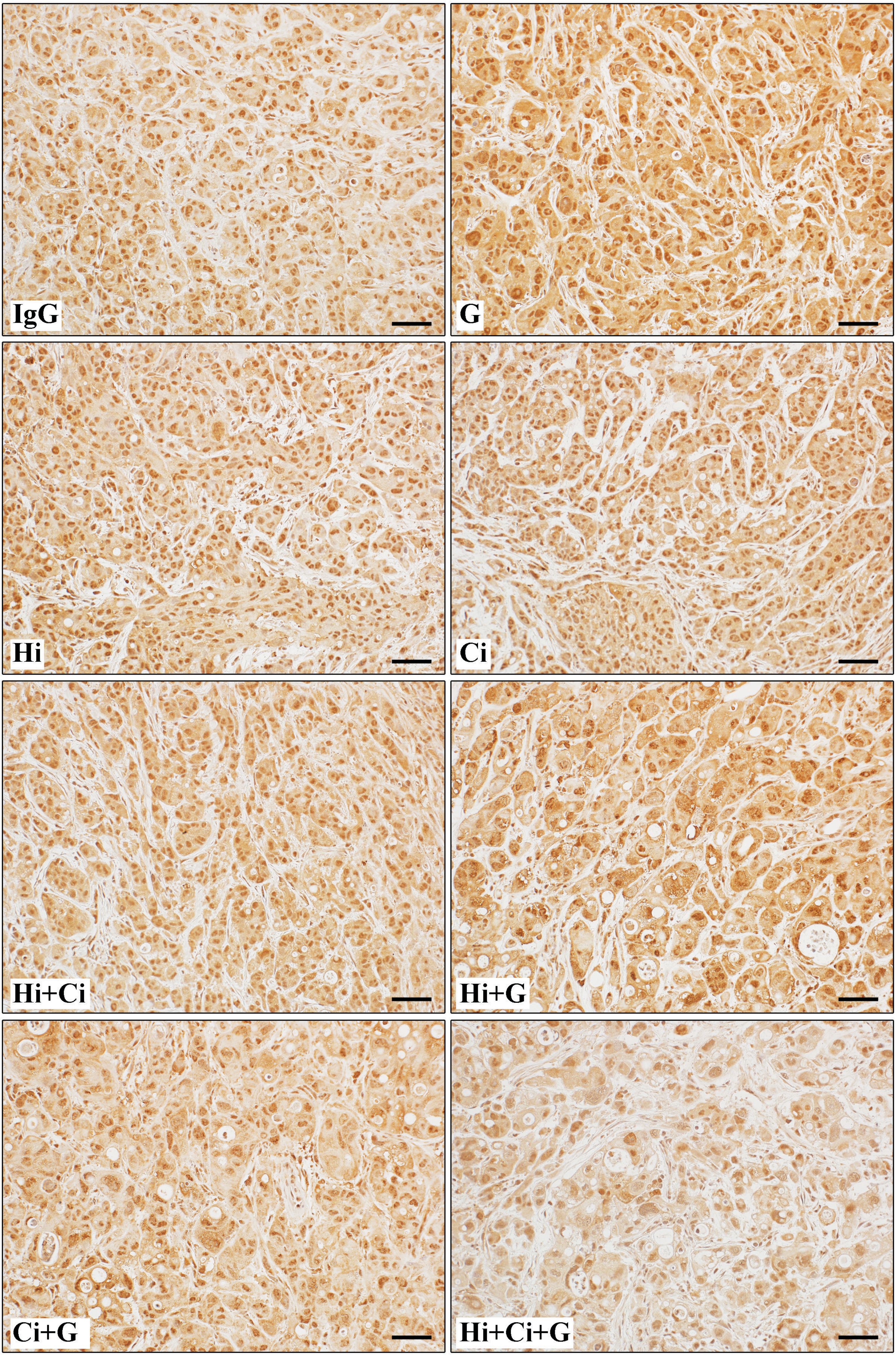

Supplementary Fig 6 A

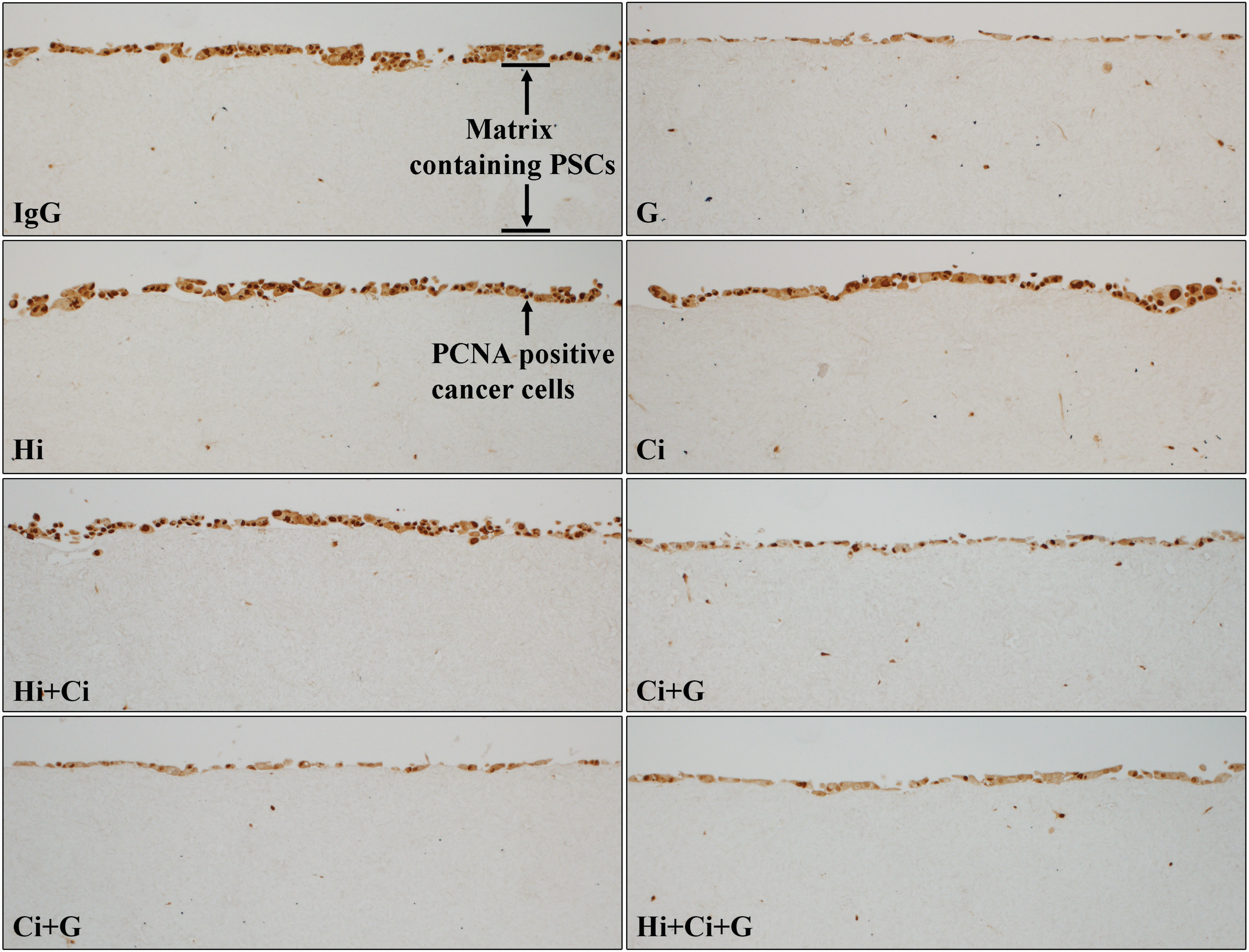

Supplementary Fig 6 B

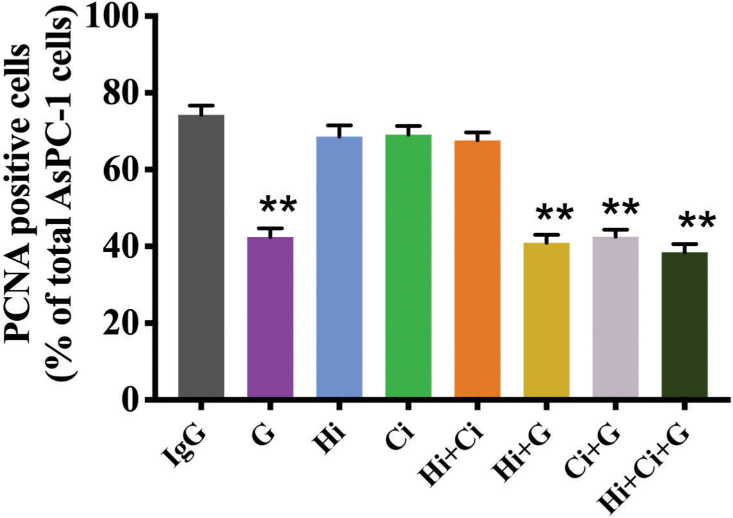

Supplementary Fig 7

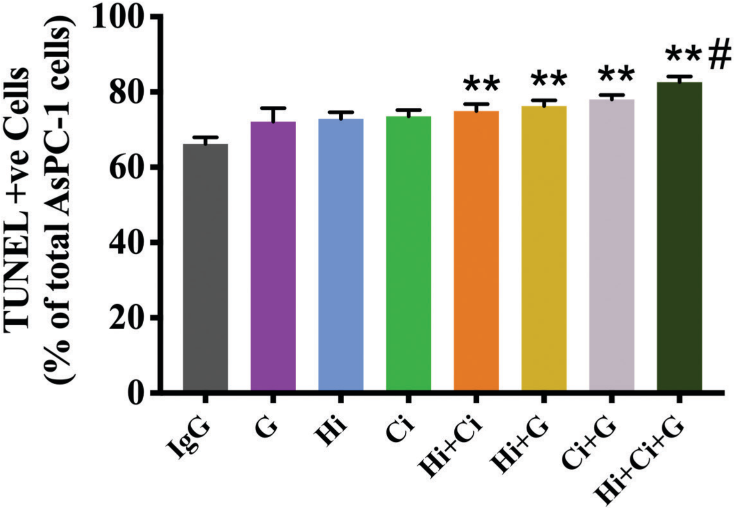

Supplementary Figure 8

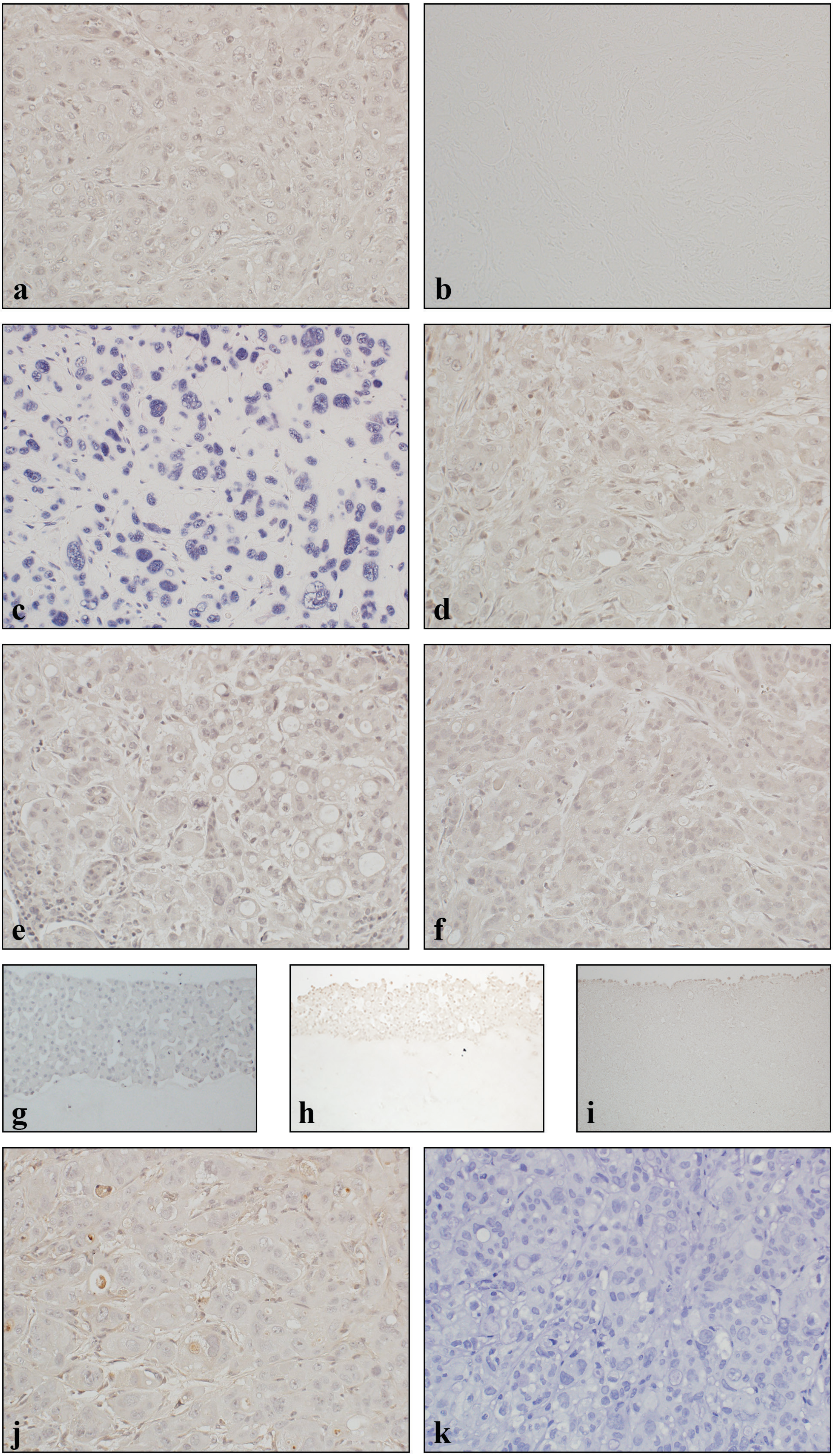

Supplement: Supplementary file 1 — Supplementary text clean ver and supplementary figures [file 41416_2020_782_MOESM1_ESM.pdf]
